# Efficient generation of transgenic cattle using the DNA transposon and their analysis by next-generation sequencing

**DOI:** 10.1038/srep27185

**Published:** 2016-06-21

**Authors:** Soo-Young Yum, Song-Jeon Lee, Hyun-Min Kim, Woo-Jae Choi, Ji-Hyun Park, Won-Wu Lee, Hee-Soo Kim, Hyeong-Jong Kim, Seong-Hun Bae, Je-Hyeong Lee, Joo-Yeong Moon, Ji-Hyun Lee, Choong-Il Lee, Bong-Jun Son, Sang-Hoon Song, Su-Min Ji, Seong-Jin Kim, Goo Jang

**Affiliations:** 1 Department of Theriogenology, College of Veterinary Medicine and the Research Institute of Veterinary Science, Seoul National University, 08826, Republic of Korea; 2 Embryo Research Center, Seoul Milk Coop, Gyeonggi-do, 12528, Republic of Korea; 3 TheragenEtex BiO Institute, Advanced Institutes of Convergence Technology, Kwanggyo Technovalley, Suwon, 16229, Republic of Korea; 4 Department of Chemistry, College of Natural Science, Seoul National University, 08826, Republic of Korea; 5 Emergence Center for Food-Medicine Personalized Therapy System, Advanced Institutes of Convergence Technology, Seoul National University, Gyeonggi-do, 16229, Republic of Korea

## Abstract

Here, we efficiently generated transgenic cattle using two transposon systems (*Sleeping Beauty* and *Piggybac*) and their genomes were analyzed by next-generation sequencing (NGS). Blastocysts derived from microinjection of DNA transposons were selected and transferred into recipient cows. Nine transgenic cattle have been generated and grown-up to date without any health issues except two. Some of them expressed strong fluorescence and the transgene in the oocytes from a superovulating one were detected by PCR and sequencing. To investigate genomic variants by the transgene transposition, whole genomic DNA were analyzed by NGS. We found that preferred transposable integration (TA or TTAA) was identified in their genome. Even though multi-copies (i.e. fifteen) were confirmed, there was no significant difference in genome instabilities. In conclusion, we demonstrated that transgenic cattle using the DNA transposon system could be efficiently generated, and all those animals could be a valuable resource for agriculture and veterinary science.

Transgenesis is an important tool to understand gene function in mammals. Based on isolation of embryonic stem cells in rodents via germline transmission, transgenic mice have been accelerated in genetic models. Unlike mice with germ-line competent embryonic stem cells, development of transgenic livestock has been hampered to date. Early studies in transgenic livestock depended on microinjecting DNA into pronuclear stage embryos. After improving DNA delivery, several transgenic techniques such as virus- or sperm-mediated gene transfer and somatic cell nuclear transfer (SCNT) with transgenic somatic cells have been applied. Recently, SCNT has been heralded as a promising approach for generating transgenic livestock. Even though abnormalities derived from SCNT are reported, a few transgenic cattle via SCNT have been generated. However, there is still low efficiency due to insufficient reprogramming and high frequency of abnormalities in the SCNT approach. An alternative approach for producing transgenic livestock is the use of viral vectors. Among several viral vectors, lentivirus-mediated gene transfer has successfully been applied to transgenic cattle. Although viral gene delivery has advantages for efficient genome integration, viral infection may cause activation of proto-oncogene, resulting in potential of tumorigenesis^1^,^2^.

Recently, in addition to simple plasmid and viral gene delivery, DNA transposons including Piggybac (PB), Sleeping beauty (SB), Tol2 or Tn5 have been successfully used for transgenesis in several studies^3^,^4^,^5^,^6^. The basic principle of transposon is that transposase recognizes transposable elements sequences (TES), cut the inside DNA of TES and paste it into the other genome position. When TES moved into another region, they preferred some specific sequences like TA and TTAA for SB and PB, respectively^7^. Furthermore, when the transposase cuts and pastes the transgene, multi-copies integration into genome is possible (i.e. over 60 copies)^8^. Also, as integration of transposon has preference for low-risk chromosomal regions such as intronic sequences^7^, it could be safer than viral gene delivery. Due to stable integration with high expression by transposon DNA delivery, transposons are applied to several species as mentioned above. Although there have been several research publications regarding transgenesis in cattle using transposon^9^,^10^, live cattle has not been produced. Here, we generated transgenic cattle using two transposons (SB and PB), which deliver ubiquitous expression, conditional expression by rox-Dre recombinase, and tissue-specific expression. Additionally, those were analyzed by next-generation sequencing (NGS) for genome integration site, number of transgenes and genomic variants (Fig. 1).

## Results

### Transgene expression in somatic cells, embryos and calf

Efficiency of transgene delivery into bovine fibroblasts was measured. Plasmid DNA (pcDNA3.1-GFP) and two transposons (SB-GFP and PB-GFP) were transfected; delivery efficiency of GFP was shown in Supplementary Figure 2. The expression ratio of GFP at 24 h, 48 h, 72 h, 96 h and 144 h after transfection without antibiotic selection in PB-GFP (12.8, 32.9, 41.7, 44.6 and 24.3%, respectively) was higher than in pcDNA3.1-GFP (1.4, 8.6, 18.7, 0.0 and 0.0%, respectively) and SB-GFP.

**Figure 2:**
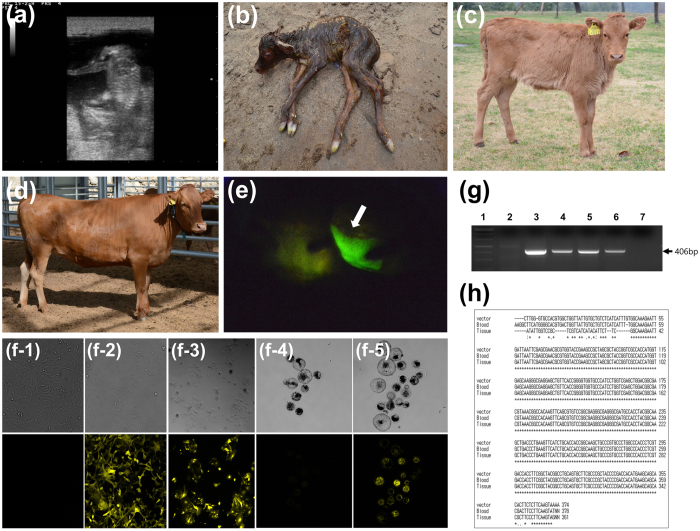
Birth of a transgenic (tg) cow with the YFP gene via *Sleeping Beauty* (SB) and its analysis. (**a**) After 60 days of embryo transfer, pregnancy was confirmed by ultrasonography. The calf was delivered without assistant (**b**) and grew to 5-months (**c**) and 16 months (**d**) old without any health issue. (**e**) When ultraviolet light was exposed to nose of tg cattle, YFP expression was found (arrow). To determine YFP expression in primary skin and endometrial cells, the cells were cultured and captured by confocal image equipment ((**f-1**) skin cells from a wild type, (**f-2**) skin cells from a tg cattle, (**f-3**) endometrial cells from a tg cattle, upper: brightness, lower: fluorescence). The primary skin cells from tg or non-tg were reprogrammed and developed into blastocysts (**f-4**) blastocysts from skin cells of non-tg cattle, (**f-5**) blastocysts from skin cells of the tg cattle; upper: brightness, lower: fluorescence). The tg integration was confirm by PCR (**g**) and sequencing (**h**). 1: Molecular maker, 2: Wild type cattle, 3: Positive control (DNAs), 4: Blood from tg cattle, 5: Ear tissues from tg cattle, 6: Placenta from tg cattle, 7: Negative controls. Gel image was cropped and original image was seen in Supplementary Figure 8.

**Figure 3:**
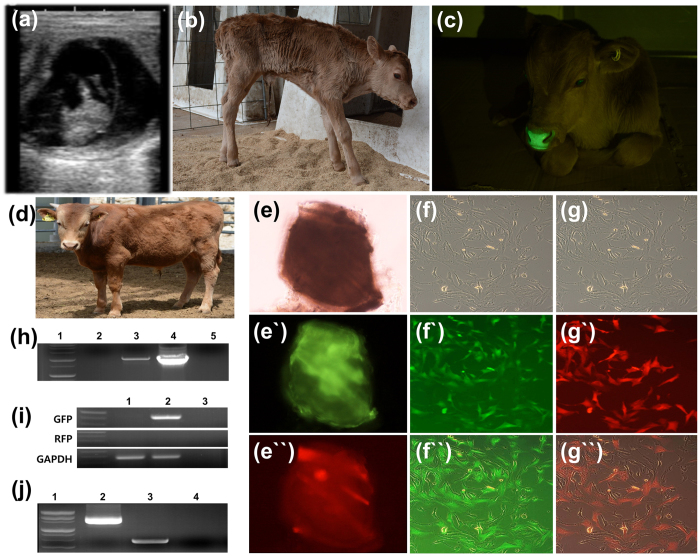
Birth of a transgenic (tg) cattle with the rox-GFP-rox-RFP gene via *Piggybac* (PB) and its analysis. (**a**) After 45 days of embryo transfer, pregnancy was confirmed by ultrasonography. (**b**) The calf was delivered without assistant. (**c**) When ultraviolet light was exposed to nose of tg cattle, GFP expression was strongly observed. And the tg cattle grew up to 12 months old without any healthy issue (**d**). To determine GFP or RFP expression in a piece of tissue or primary skin cells via recombination, the tissue and cells were cultured and transfected with Dre recombinase mRNA by nucleofection ((**e**) a piece of tissue from tg cattle-brightness, (**e`**) before Dre recombinase transfection (GFP), (**e``**) after Dre recombinase transfection (RFP)). The primary skin cells from the tg cattle were isolated, cultured and transfected with Dre recombinase mRNA. Before transfection, only GFP expression was observed, RFP expression were observed via GFP gene excision by recombination ((**f**–**f``**) before transfection brightness, fluorescence, and merged, respectively; (**g**–**g``**) after transfection brightness, fluorescence, and merged, respectively). The transgene integration and recombination were confirmed by genomic DNA PCR ((**h**) 1: Molecular maker, 2: Wild type cattle, 3: Blood from tg cattle, 4: Positive control (DNAs), 5: Negative control) and RT-PCR ((**i**) 1: Wild type cattle, 2: cDNA from tg cattle, 3: Negative control). After Dre recombinase transfection, GFP excision was confirmed by genomic DNA PCR ((**j**) 1: Molecular marker, 2: Before transfection, 3: After transfection, 4: Negative control). Gel image was cropped and original image was seen in Supplementary Figure 8.

**Figure 4:**
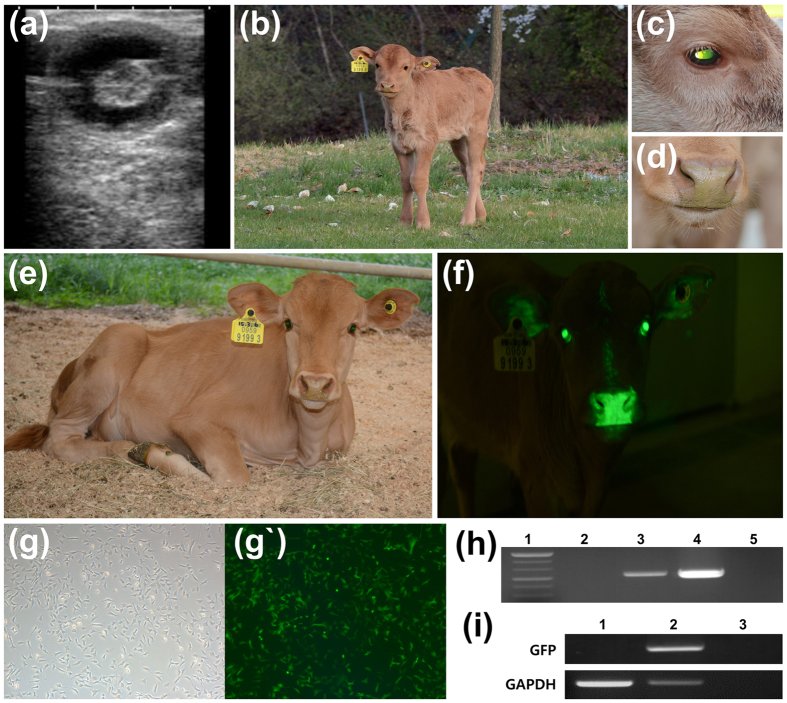
Birth of a transgenic (tg) cattle with the pβ-casein-hIL2-pCAG-GFP gene via *Piggybac* (PB) and its analysis. (**a**) After 45 days of embryo transfer, pregnancy was confirmed by ultrasonography. (**b**) The calf was delivered without any assistance and grew up to 2 months. Analyzing the calf without ultraviolet light, GFP expression was observed in the eyes (**c**) and nose (**d**). The tg cattle have been grown to 5 months old without any health issue (**e**). When ultraviolet light was exposed to the head, GFP expression was strongly observed (**f**). To know GFP in skin cells, the primary skin cells from the tg cattle were isolated and cultured. In over 99% of cells, GFP expression were observed ((**g**) brightness; (**g`**) fluorescence). The transgene integration was confirmed by genomic DNA PCR ((**h**) 1: Molecular maker, 2: Wild type cattle, 3: Blood from tg cattle, 4: Positive control (DNAs), 5: Negative control) and RT-PCR using primary cells ((**i**) 1: cDNA from Wild type cattle, 2: cDNA from tg cattle, 3: Negative control). Gel image was cropped and original image was seen in Supplementary Figure 8.

SB-YFP and SB100X were microinjected into 191 fertilized embryos. Twenty blastocysts were formed and one of them expressed YFP without mosaicism. PB-rox-GFP-rox-RFP and transposase were microinjected into 560 fertilized embryos, and 93 blastocysts (11 GFP expression) were formed. After microinjecting PB-pβ-casein-hIL2-pCAG-GFP and transposase into 4033 fertilized embryos, 49 of 779 blastocysts expressed GFP. Selected blastocysts with ubiquitous expression were transferred into 17 recipients. A total of nine recipients were pregnant and nine transgenic calves were naturally delivered (Figs. 2, 3, 4; Supplementary Figure 4). One died due to respiratory distress with delayed delivery (Supplementary Figure 3), another was suffered from severe diarrhea, and died one month later (Table 1).

Integration of transgene confirmed by PCR (Figs. 2, 3, 4; Supplementary Figure 4) using genomic DNAs. Primary cells were isolated from all the transgenic calves and expressed GFPs. Number of fluorescence-positive cells was calculated and summarized (Table 1). After primary cells from SNU-PB-1 were transfected with mRNA of Dre recombinase, recombination reaction was confirmed by RFP expression and genomic PCR amplification (Fig. 3).

Light or strong green color (fluorescent response) in some organs (the hooves, nose, eyes, lips and tongues) were observed under normal lights in some transgenic cattle (Fig. 4 and Supplementary Figure 4). Among the organ, the strongest expression was founded in the eyes (Figs. 3 and 4; Supplementary Figure 4).

### Copy number and integration site

To detect integration events of transgene, single-nucleotide variants (SNV), structural variation (SV), and copy number variations (CNV), whole genome sequence from three transgenic and wild type cattle blood samples were analyzed. On average, more than 60 giga base pairs (Gbp) per sample were produced (Table 2). Based on the sequencing quality metrics, we estimated about 16-fold coverage of whole genome of cattle with the quality passed and aligned paired-end reads. The average mapping rate to the cow reference genome (UMD3.1) was over 99.73% (Table 2).

For integration site and copy number, all the transgene sites were found by the Integrative Genomics Viewer (IGV) program (https://www.broadinstitute.org/igv/, Broad Institute) and confirmed manually by PCR with endogenous and exogenous specific primers (Supplementary Table 1). The YFP gene (SNU-SB-1) was integrated in chromosomes 4, 21 and 26. One transgene was integrated in intron between exons 1 and 2 at chromosome 4, locus designed for GNAI1 (Genbank assess NM_174324.2). To evaluate transcripts of GNAI1, RT-PCR was performed and its expression was not shown to be affected (Supplementary Figure 5).

**Figure 5:**
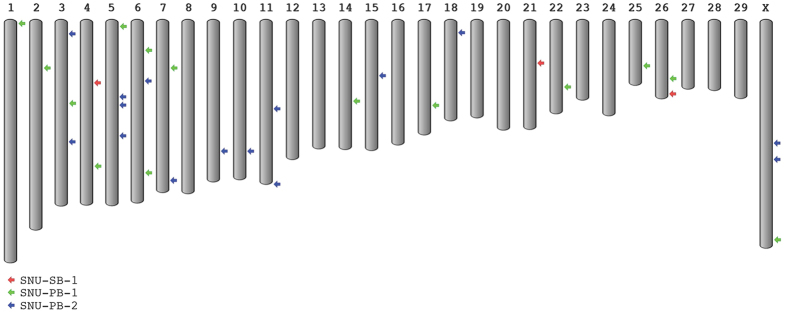
Analysis of transgene integration sites in cattle showed that shared integration of site and unique site existed as integration event. Each index color showed individual sample.

The rox-GFP-rox-RFP gene (SNU-PB-1) was integrated in chromosomes 1, 2, 3, 4, 5, 6 (two sites), 7, 14, 17, 22, 25, GJ0599801.1, 26 and X. The pβ-casein-hIL2-pCAG-GFP gene was integrated in chromosomes 3 (two sites), 5 (three sites), 6, 7, 9, 10, 11 (two sites), 15, 18 and X (two sites). All the integrated sites including exact position and 5′-, 3′- flanked genes were summarized Table 3 and illustrated in Fig. 5.

### Identification of transgenic variants compared to wildtype

**Figure 6:**
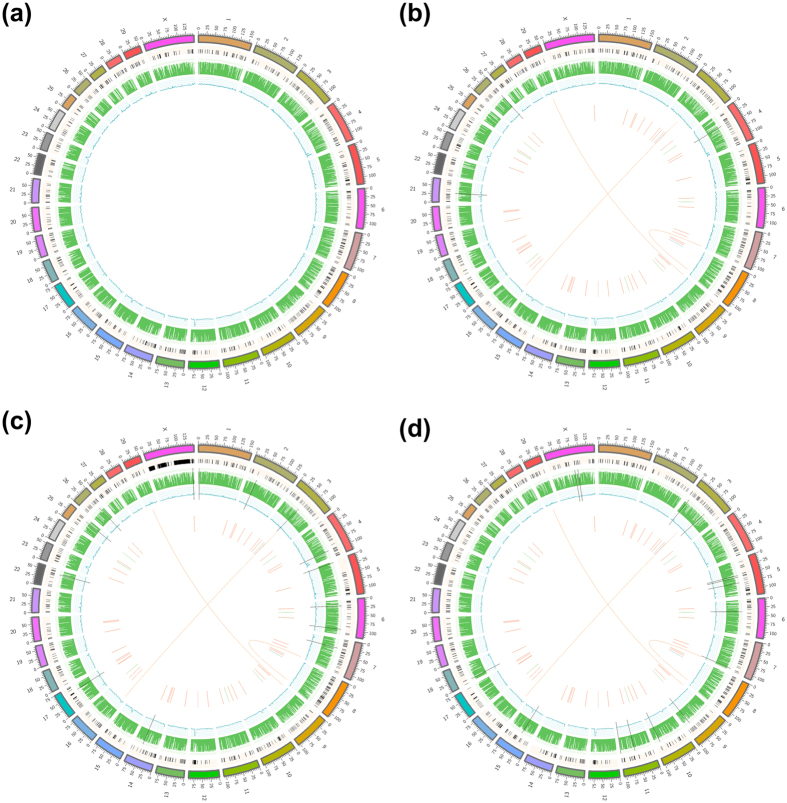
Overview of genomic variation in cattle. Reference chr (stands for chromosome) containing from chr1 to X chr is colored in a variety of different colors in peripheral boxes. And, copy number variation (CNV), coverage and histogram indicating SNP density of sample plotted per 10-kb windows are colored in black, green and blue colors, respectively. Structural variations (SVs) including deletion (red), translocation (orange), inversion (green) and duplication (blue) are indicated by lines and links. Black lines passed through the coverage (green) and the histogram (blue) refers to the integration sites of transgenes in the reference chr. (**a**) Wild type, (**b**) A transgenic cattle using sleeping beauty transposon (SNU-SB-1), (**c**) A transgenic cattle using *piggybac* (SNU-PB-1), (**d**) A transgenic cattle using *piggybac* (SNU-PB-2).

In transgenic and wild type, overall, about 8.1 million SNVs and 1.0 million insertions and deletions (Indels) were identified (Table 4). Using this data, we investigated the transgenic-specific SNV. The number of transgenic-specific SNV, as “high impact” by SnpEff software (http://snpeff.sourceforge.net/, version 4.2) were 315 (Table 5; Supplementary Table 2). Furthermore, we also identified the transgenic-specific SV and CNV were 65 and 38, respectively (Supplementary Tables 3 and 4). The SV event was consisted of 49 deletions, 2 duplications, 8 inversions and 6 translocations. In the case of CNVs, there were 33 gains and 5 losses. In our analysis, SNP density of chromosome 12 and 23 in all samples were very high compared to other chromosomes (Fig. 6).

### Telomere length analysis

Telomeric sequences (TTAGGG) were measured by analysis software, used as in a previous study^11^. Its length was described in Table 6 (SNU-SB-1: 6.59, SNU-PB-1:7.26, SNU-PB-2: 6.98, Wild type: 5.69).

### Disruption of GFP and Knock-in

Transgene integration positions in the transgenic cattle were considered at the safe target region because they have grown up without health issues to date. Thus, we transfected guide RNA endonuclease for the GFP as a previous study^12^ and donor knock-in DNAs together into the primary cells from SNU-PB-2. After transfection, during three days, the cells were isolated with antibiotic selection, puromycin. On 10 days post-transfection, we found the several colonies without GFP expression only in GFP guide RNA/Cas9 + Donor DNAs group. In the other groups (control, GFP guide RNA/Cas9 and Only Donor group; Supplementary Figure 6), all of the cells were dead.

### Transgene detection in Germ cells

In one cattle (SNU-SB-1, female), we performed superovulation, artificial insemination and embryo collection. We failed to collect viable fertilized embryos. Nine unfertilized oocytes were collected and the transgene were detected by genomic PCR. When collecting the embryos in uterus, some tissues from uterine epithelium were isolated and cultured. All uterine epithelial cells expressed YFP protein (Supplementary Figure 7).

## Discussion

Transgenic cattle in agriculture fields have been of interest due to basic embryology and genetic models. Although several trials to generate transgenic cattle have been carried out, the number of live transgenic cattle and germ line transmission of transgene into NGS have been hampered to date. While live transgenic cattle and germ line transmission using lentiviral-mediated transgenesis has been applied successfully^13^, the issue that viral gene delivery may cause oncogenic activation remains. As an alternative approach, nuclear transfer is considered. It has several disadvantages such as very low efficiency, abnormalities and sudden deaths. To overcome those issues on transgenic cattle, here, we reported efficient production of transgenic cattle using the transposon system. Furthermore, transgene integration and genome variants were analyzed by NGS for genomic stability of transgenic cattle.

DNA transposon is well established to generate target gene overexpression in rodents, particularly gene function via mutagenesis^14^ or cancer study^8^. Additionally, in human cells, SB or PB delivery have been used for gene therapy^15^,^16^,^17^. Unlike mice, development of gene function via transgene delivery in mutant live offspring in livestock have been slow to garner to attention due to low efficiency or severe mosaicism of microinjection and nuclear transfer^18^ at greater costs. Recently, SB transposon has been successfully applied to generate transgenic pigs and its germline transmission^19^. However, progress of transgenic cattle has relatively been very slow due to long-term gestational periods (around 280 days) and single calf pregnancy even though several transgenic cattle using nuclear transfer has been born with low efficiency. Here, we introduced two DNA transposons (SB and PB) for generating transgenic cattle. While two transposons were microinjected into *in vitro* fertilized bovine embryos, the efficiency of transfections was tested to see which transposon could be better. In the test, we found that PB transposon was much higher efficiency than SB; thus, most microinjection into fertilized embryos was carried out by PB transposon.

We produced three kinds of transgenic cattle. First, a transgenic cattle expressing transgene (YFP) under the ubiquitous promoter was born via a SB transposon method (Fig. 2). Second, a transgenic cattle with conditional gene expression by Dre recombinase was born via a PB transposon. In this cattle, ordinarily GFP transgene was expressed in the whole body. Furthermore, after Dre recombinase treatment, GFP gene excision occurred and sequentially RFP gene was expressed (Fig. 3). Lastly, several transgenic cattle with tissue specific promoter (beta-casein)-human gene (IL2) with reporter gene (GFP) via PB transposon were born (Fig. 4). During this study, we did not find either any miscarriage or stillbirths in recipient cow after diagnosing pregnancy or health problems in growing cattle.

Moreover, we wondered that the transgene expression was in germ cells because the first transgenic cattle (SNU-SB-1) have only reached puberty. After superovulation, artificial insemination, and embryo collection, we did not find viable fertilized embryos. Nine unfertilized oocytes were collected, and transgene expression in these oocytes was detected and confirmed by sequencing (Supplementary Figure 6). Additionally, the uterine epithelial cells, which were collected by uterine flushing, expressed 100% the YFP protein (Supplementary Figure 6). Because the second transgenic cattle will be in puberty and fertile, we are planning to produce the calf between the first (female) and second (male) transgenic cattle by natural breeding. During the submission and review, natural breeding was carried out and the transgenic cattle (SNU-SB-1: female) was pregnant. On coming end of June at this year, the calf will be delivered and analyzed by PCR and fluorescence expression for germline transmission. Furthermore, the semen from the second transgenic cattle (SNU-PB-1: male) were collected, frozen and used for *in vitro* fertilization. As expected, the GFP expressing blastocysts were observed. In the future, when the recipients are ready, the blastocysts with GFP expression will be transferred for producing a female and a male calf. The calves will be bred for producing a homozygous offspring.

One of the most important issues in transgenic animals is integration-number, -site and expression of the transgene because it may affect the lethality or gene silencing^20^,^21^. Theoretically, when target gene by transposons (jumping gene) move into another site, it has moved into its preference sequences (TA for SB and TTAA for PB)^7^. In this study, to confirm transposon preference and genome instability (copy number variation, structure variation and telomere lengths), the genome from blood of these transgenic cattle was analyzed by NGS. As expected, the genes by SB and PB were integrated into TA or TTAA position, respectively (Table 2). While a few transgene copies were inserted intron of coding gene, most transgene were integrated in non-coding region. Even though transgene were integrated in intron of coding gene (exons), its transcriptional expression was not changed (Supplementary Figure 5).

We assume that these transposon integrations may not affect the normality of transgenic cattle in our study even though high number of transgene integration (over than 10 copies) were found. Thus, we believed that the transgene integration sites could be used as the target region (*safe harbor regions*, such as mROSA26 and hAAVS1) for another useful protein expression using genome-editing technologies. For this approach, RNA-guided endonuclease for GFP was applied and all the GFP regions were disrupted. Furthermore, recombination knock-in cassette using donor DNAs were integrated in GFP target site. In future, we will add a gene of interest into the target locus by Cre-recombinase-based exchange and used as the donor cells for producing cloned cattle.

In the previous reports on transgenic animals or plants generated by transposons and plasmids, they did the integration or expression based on conventional PCR approaches^5^,^22^,^23^. Its disadvantage is to find out only amplified products with primer conditions, indicating that not all the transgenes can be identified. Transgene insertion site is not typically characterized because traditional methods for transgene insertion site discovery are either expensive and/or offer low resolution (DNA FISH) or are complicated by the multi-copy nature of the inserted sequences (inverse PCR). However, whole genome sequencing enables us to find out all the integration details with high specificity at single-nucleotide resolution and also provided information on the chromosomal location and transgene copy number^24^,^25^. Indeed, in our study NGS analysis provided transgene integration number and position with single nucleotide resolution. Furthermore, we hypothesized that as the transposon moved initial site into another position, the genome variants such as SNP, SV, CNV and telomere lengths might be affected in these transgenic cattle. When we analyzed the genome variants in 5′- and 3′-region (1 kbps) of the transgene integration positions, there were no significant genomic variants. On chromosomes 12 and 23, on the other hand, we found high variable regions as previously reported^26^. The result indicated that it was breed-specific characteristics, not transgenic cattle.

Using NGS analysis, relative telomere lengths, which is co-related to age of individual were measured on the transgenic cattle to know if transposition of transgenes might affect the telomere length or not. Although there were no considerable changes in telomere length, only one transgenic cattle showed shorter size telomere compared to other individuals. To figure out whether senescence changes could be identified for the transgenic cattle, its development to adult will be monitored.

In conclusion, the data demonstrated that, for the first time, we generated several transgenic cattle efficiently using the DNA transposon delivery system and identified integrated number, integration position, genomic variants and telomere lengths by the NGS approach. They have grown up to date without any health issue and breeding. We suggest that those transgenic cattle could be valuable resources for bio-agricultural science.

## Materials and Methods

### DNA preparation

DNA preparation for SB containing yellow fluorescence protein (YFP) and SB100X transposase were reported previously. The transposase plasmids for SB (pCMV(CAT)T7-SB100X) and PB (pCy43) were purchased from Addgene (http://www.addgene.org, Plasmid#34879) and provided by Sanger Institute (Hinxton, UK). Rox-GFP-polyA-rox and RFP were amplified by gateway PCR cloning (MultiSite Gateway^®^ Pro Plus, Invitrogen, 12537100, Life Technologies, Carlsbad, CA, USA) and inserted into final expression vector, PB-CAG (http://www.addgene.org/, #20960). Beta-Casein promoter and hIL2 cDNAs were amplified by PCR and inserted into PB-GFP by Infusion Cloning (In fusion HD cloning kit, Clontech, 639644, California, US). All the DNA vectors used in this study were illustrated in Supplementary Figure 1.

### *In vitro* maturation, fertilization and culture of bovine immature oocytes

#### Oocyte collection and *in vitro* maturation (IVM)

Ovaries were collected from a local abattoir into saline at 35 °C and transported to the laboratory within 2 h. Cumulus-oocyte complexes (COCs) from follicles 2–8 mm in diameter were aspirated using an 18 gauge needle attached to a 10 ml disposable syringe. COCs with evenly-granulated cytoplasm and enclosed by more than three layers of compact cumulus cells were selected and washed three times in HEPES-buffered tissue culture medium-199 (TCM-199; Invitrogen, Carlsbad, CA, USA), supplemented with 10% FBS, 2 mM NaHCO_3_ (Sigma–Aldrich Corp., St. Louis, MO, USA), and 1% penicillin–streptomycin (v/v). For IVM, COCs were cultured in four-well dishes (30–40 oocytes per well; Falcon, Becton-Dickinson Ltd., Plymouth, UK) for 22 h in 450 μL TCM-199 supplemented with 10% FBS, 0.005 AU/ml FSH (Antrin, Teikoku, Japan), 100 μM Cysteamine (Sigma-Aldrich), and 1 μg/ml 17β-estradiol (Sigma–Aldrich) at 39 °C in a humidified atmosphere of 5% CO_2_.

#### Sperm preparation, *in vitro* fertilization (IVF) and *in vitro* culture of embryos (IVC)

Motile spermatozoa were purified and selected using the Percoll gradient method^27^. Briefly, spermatozoa were selected from the thawed semen straws by centrifugation on a Percoll discontinuous gradient (45–90%) for 15 min at 1500 rpm. The 45% Percoll solution was prepared with 1 mL of 90% Percoll (Nutricell, Campinas, SP, Brazil) and 1 mL of capacitation-TALP (Nutricell). The sperm pellet was washed twice with capacitation-TALP by centrifugation at 1500 rpm for 5 min. The active motile spermatozoa from the pellet were used for insemination of matured oocyte (At 24 h of IVM). Oocytes were inseminated (day 0) with 1–2 × 10^6^ spermatozoa/mL for 18 h in 30 μL microdrops of IVF-TALP medium (Nutricell) overlaid with mineral oil at 39 °C in a humidified atmosphere of 5% CO_2_. Presumptive zygotes were denuded and cultured in two-step chemically defined culture medium overlaid with mineral oil (Sigma–Aldrich)^28^. All incubations were done at 39 °C in an atmosphere of 5% O_2_, 5% CO_2_ and 90% N_2_. Cleavage rates were recorded on Day 2 and embryonic development was monitored according to the stages of the International Embryo Transfer Society (IETS).

### Microinjection

Transposon DNAs were microinjected into the cytoplasm by microinjector machine (Femtojet^®^, Eppendorf, Germany) after removing the cumulus cells of fertilized oocytes. Amount of injected DNAs was 100 ng/mL (1:1 ratio of transposon and transpoase). After 7 days, GFP expressing pre-implantational stage embryos were chosen and transferred into the surrogate cow.

### Embryo transfer and pregnancy diagnosis

All experiments with live animals were performed in accordance with the relevant laws and institutional guidelines of Seoul National University and Seoul Milk Coop, and institutional committees of Seoul Milk Coop have approved the experiments. A GFP-expressing blastocyst in PBS supplemented with 20% FBS was transferred to the uterine horn of each recipient cow by a transcervical method on Day 7 (estrus = Day 0 = day of fusion) by non-surgical approach. In order to determine embryo survival and pregnancy, cows were examined by rectal palpation and ultrasonography on Day 45 post estrus. Pregnant cows were monitored by rectal palpation and ultrasonography at regular intervals thereafter.

### Genomic DNA sample preparation

Genomic DNA was extracted from blood or primary cells with DNA extraction kit, following the manufacturer’s protocol. Genomic DNA was analyzed by Qubit fluorometer dsDNA assay Kit (Invitrogen, CA) as well as Infinite F200 Pro NanoQuant (TECAN, Männedorf) to verify the quality (O.D. 260/280 ratio is 1.8–2.0 and O.D. 260/230 ratio greater than 1.6) and quantity (1 ug for library construction).

### Library construction and sequencing

One μg of genomic DNA for a 350 bp insert size was fragmented using a Covaris S2 Ultrasonicator. DNA sequencing libraries were constructed using the TruSeq DNA PCR-Free Sample Preparation Kit from Illumina (San Diego, CA). They were prepared according to the manufacturer protocol by eliminating PCR amplification steps to removes typical PCR-induced bias and streamlines. The final library size and quality were evaluated electrophoretically with an Agilent High Sensitivity DNA kit (Agilent Technologies, Santa Clara).

Sequencing was done on Illumina HiSeq 2500 using the TruSeq Paired End Cluster Kit v3 and the TruSeq SBS Kit v3-HS (FC-401-3001), generating 2 × 100  bp reads at TheragenEtex Bio Institute, Korea. Image analyses were performed using the HiSeq control software (Version 2.2.58). Raw data was processed and base calling performed using the standard Illumina pipeline (CASAVA version 1.8.2 and RTA version 1.18.64).

### Sequencing data quality control

Over about four hundred million pass-filter reads were generated per each sample. Quality control analysis of the sequencing reads was conducted using the FastQC software^29^ and In-house script. During data analysis, the raw reads obtained from sequencing were trimmed for low quality ends with the Sickle software (version 1.33)^30^, using a Phred quality threshold of 20. All reads shorter than 50 bp after the trimming were discarded.

### Read mapping and analysis

All of the filtered sequencing reads were then mapped to the reference Bos Taurus genome sequence (UMD 3.1, http://asia.ensembl.org/Bos_taurus/Info/Annotation) and the transgene sequence at once using Burrows-Wheeler Aligner (BWA, version 0.7.5a)^31^. To avoid overweighting of some genomic positions caused by inhomogenoerous PCR amplifications, we removed duplicated reads with the MarkDuplicate subroutine (Picard, version 1.128).

### Variant analysis

Multi-sample calling of single-nucleotide variants (SNV) and indels was performed on processed, sample-level BAM files with the GATK Unified Genotyper^32^. After multi-sample calling, variants were first filtered for confident calls using a quality score cutoff of 30. The SnpEff software^33^ together with the UMD 3.1/bosTau Ensembl annotation was used to predict the functional effects of the variants detected.

### Identification of copy number variations (CNVs) and structural variations (SVs)

To identify copy number changes in cattle, we used the Control-FREEC software^34^. FREEC calculates ploidy for the regions of interest as the copy number value in each 50 kb window in the region of interest after GC content read count normalization, given a normal autosomal ploidy of 2. SVs (deletions, tandem duplications, inversions and translocations) called at nucleotide resolution with split-read support using Delly software^35^ that uses diploid genotype likelihoods and the best likelihood determines the final genotype. We use the 3 criteria of the precision filter as follow. First, we use the PRECISE/IMPECISE creteria. PRECISE are structural variant calls at nucleotide resolution with split-read support. we select only PRECEISE structural variant call. Second, we select >=20 the paired-end support of the structural variant. Third, the mean mapping quality (MAQ) has to be >=60.

To compare calls between transgenic and wild type, we used bedtools software^36^ intersect requiring 80% reciprocal overlap (-r -f 50). If this condition is satisfied more than 2 transgenic, this SV consider the same things. And then this compared to wild type for identifying transgenic-specific SVs. Transgenic-specific CNV was called in the same way.

### Transgene insertion site detection

With mapping data BAM (aligned format) generated by BWA, we analyzed the insertion site of transgene. BWA meant that some nucleotides at either extreme of the read could be omitted (that is, “soft trimmed” or “soft clipped”) as determined by a Smith-Waterman like scoring scheme. By checking the mapped pattern of soft-clipped sequence, we inferred the insertion candidate sites. In parallel, we also used Delly to detect whether genome structural variation can be a candidate for the insertion site of transgene. Lastly, the candidate sites were also manually inspected using the IGV software.

### Calculation telomere length using whole genome sequence

Whole genome data are mined for reads that are rich in telomere sequence, and relative length is determined. Using TelSeq^11^, we examined the frequency of reads from transgenic and control with different number of copies of TTAGGG.

### PCR and RT-PCR

To confirm expression of mRNA or DNA integration, PCR and RT-PCR were carried out. Genomic DNA was extracted from blood or cells using DNA extraction kit (DNeasy Blood&Tissue kit 69506, Qiagen, Limburg, Netherlands). Total RNAs were extracted using an RNA extraction kit (Easy spin total RNA extraction kit, Cat no. 17221, iNtRON, Seongnam-si, Korea). One ug total RNAs were used for synthesizing cDNA using a cDNA synthesis kit (RNA to cDNA EcoDry™ Premix Kit, PT5153-2, Clontech, California, US). Amplification of the target DNA using specific PCR primers was performed by PCR machine (Eppendorf Vapo Protect Mastercycler, Eppendorf, Germany).

### Disruption of GFP and Knock-In

In a transgenic cattle, GFP gene was disrupted by RNA-guided endonuclease (CRISPR/Cas9) as previously reported^12^. As briefly, primary cells from a transgenic cattle (SNU-PB-2) were transfected with plasmid DNAs (Cas9 with CMV promoter, single guide RNA for GFP with U6 promoter (Toolgen, Seoul, Republic of Korea), donor DNAs for Knock-In; Supplementary Figure 6) using Nucleofactor technology (Neon^®^, Invitrogen; program #16). After transfection, the fibroblasts were cultured with 4 ug/mL, Puromycin (GIBCO) for 3 days. After replacing the media with fresh culture media, the cell were cultured for an additional 10 days to find out out-growing colonies.

## Additional Information

**How to cite this article**: Yum, S.-Y. *et al*. Efficient generation of transgenic cattle using the DNA transposon and their analysis by next-generation sequencing. *Sci. Rep.* **6**, 27185; doi: 10.1038/srep27185 (2016).

## Supplementary Material

Supplementary Information

Supplementary Table 1

Supplementary Table 2

Supplementary Table 3

Supplementary Table 4

## Figures and Tables

**Figure 1:**
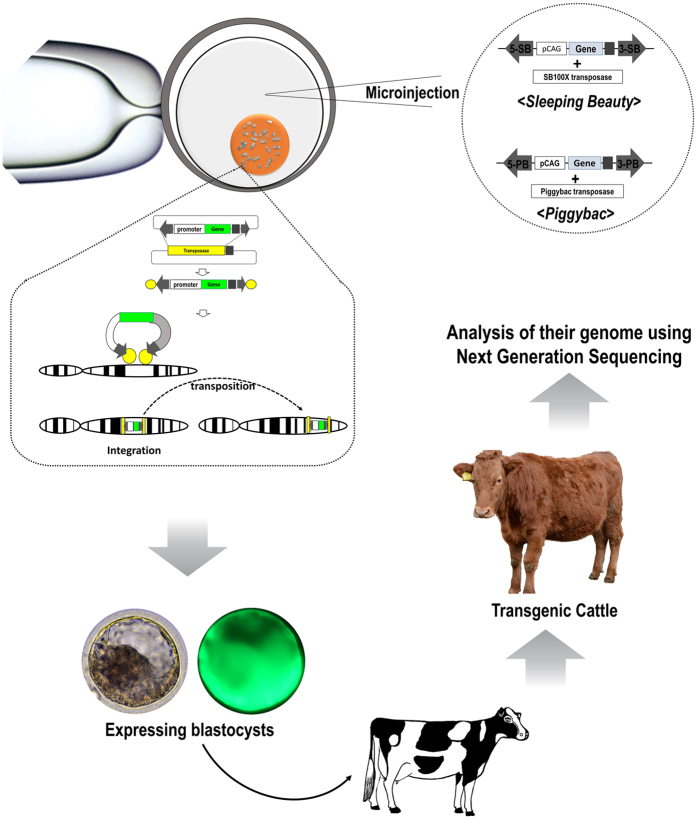
Illustration of this experiments. Transposon DNAs were mincroinjected into fertilized embryos and the blastocysts with transgene were transferred into recipient cow. Some transgenic cattle were born, and NGS analysis was used for their genome variants.

**Table 1:** Summary of birth of transgenic cattle using microinjection of DNA transposon.

I.D.	DNA	Transposon	Breed	Gender	Expression*	Age
SNU-SB-1	SB-pCAG-YFP	Sleeping Beauty	Beef cattle (Han-Woo)	Female	100%	26 months
SNU-PB-1	PB-pCAG-rox-GFP-rox-RFP	Piggybac	Beef cattle (Han-Woo)	Male	99%	19 months
SNU-PB-2	PB-pβ-casein-hIL2-pCAG-GFP	Piggybac	Beef cattle (Han-Woo)	Female	99%	12 months
SNU-PB-3	PB-pβ-casein-hIL2-pCAG-GFP	Piggybac	Beef cattle (Han-Woo)	Male	77%	Died after birth
SNU-PB-4	PB-pβ-casein-hIL2-pCAG-GFP	Piggybac	Beef cattle (Han-Woo)	Male	96%	10 months
SNU-PB-5	PB-pβ-casein-hIL2-pCAG-GFP	Piggybac	Beef cattle (Han-Woo)	Male	26%	Died due to severe diarrhea
SNU-PB-6	PB-pβ-casein-hIL2-pCAG-GFP	Piggybac	Dairy Cattle (Holstein)	Male	56%	8 months
SNU-PB-7	PB-pβ-casein-hIL2-pCAG-GFP	Piggybac	Dairy Cattle (Holstein)	Female	91%	8 months
SNU-PB-8	PB-pβ-casein-hIL2-pCAG-GFP	Piggybac	Dairy Cattle (Holstein)	Male	53%	8 months

^*^Expression percentage was calculated by ration of GFP positive cells in primary cells.

**Table 2:** Summary of sequencing results for transgenic and wild type cattle.

I.D. (DNA; Resources)	Reads	Mapped Reads	Raw coverage#1	Analysis coverage#1
Wild type (No transgene; Blood)	542,361,156	540,861,561 (99.72%)	22.86	16.06
SNU-SB-1 (SB-pCAG-YFP; Blood)	532,811,029	531,298,284 (99.72%)	22.44	15.80
SNU-PB-1 (PB-pCAG-rox-GFP-rox-RFP; Blood)	537,542,347	536,035,830 (99.72%)	22.86	15.78
SNU-PB-2 (PB-pß-casein-hIL2-pCAG-GFP; Blood)	537,419,066	536,196,898 (99.77%)	21.64	15.88

^#1^Raw coverage corresponds to the sequencing reads generated from machine. Analysis coverage is calculated from quality filtered reads and this dataset is used for insertion site discovery.

**Table 3:** All integration sites in transgenic cattle.

I.D	No.	Chromosome	Insertion site	Orientation	Overlapping gene	Location	5′ gene	3′ gene
SNU-SB-1	1	4	41,232,050–41,232,051	Reverse	GNAI1	E1–2 intron	GNAT3	PHTF2
	2	21	28,416,682–28,416,683	Forward	–		TRPM1	APBA2
	3	26	48,405,454–48,405,455	Forward	–		MKI67	EBF3
SNU-PB-1	1	1	2,651,736–2,651,737	Reverse	–		MIS184	HUNK
	2	2	31,593,723–31,593,724	Forward	–		SLC38A11	COBLL1
	3	3	54,580,493–54,580,494	Forward	–		GBP5	GBP4
	4	4	95,433,564–95,434,563	Forward	–		TSGA13	MKLN1
	5	5	4,588,449–4,588,450	Reverse	–		ATXN7L3B	CAPS2
	6	6(1)	20,085,913–20,086,912	Forward	–		DKK2	GIMD1
	7	6(2)	99,730,977–99,731,976	Reverse	PLAC8	E3–E4 intron	PLAC8	COQ2
	8	7	31593691 to 31593728	Forward	–		ERAP2	LNPEP
	9	14	53,149,061–53,149,062	Reverse	–		CSMD3	CSMD3
	10	17	55,906,674–55,907,673	Forward	KDM2B	E4-E5 intron	ORAI1	RNF34
	11	22	43,933,057–43,933,058	Forward	SLMAP	E1–2 intron	bta-mir-2370	DENND6A
	12	25	30,150,644–30,151,643	Forward	–		AUTS2	ENSBTAG00000047342
	13	26	38,489,750–38,490,749	Reverse	–		EMX2	RAB11FIP2
	14	GJ059980.1	21,074–22,073	Forward	–		–	–
	15	X	143,271,631–143,272,662	Forward	UTY	E3-E4 intron	WWC3	DDX3Y
SNU-PB-2	1	3(1)	9,538,861–9,539,321	Forward	ENSBTAG00000005796	E1-E2 intron	PEX19	PEA15
	2	3(2)	79,749,737–79,750,160	Forward	MGC137454	E2-E3 intron	PDE4B	OB-R
	3	5(1)	50,479,229–50,479,653	Reverse	–		TMEM5	AVPR1A
	4	5(2)	55,868,825–55,869,356	Forward	–		XRCC6BP1	CTDSP2
	5	5(3)	75,829,995–75,830,420	Reverse	–		MPST	KCTD17
	6	6	40,200,341–40,200,867	Reverse	–		LCORL	SLIT2
	7	7	104,834,424–104,835,058	Reverse	–		C7H5orf30	NUDT12
	8	9	85,788,192–85,788,618	Forward	–		STXBP5	SAMD5
	9	10	85,854,063–85,854,558	Forward	LIN52	E5-E6 intron	ALDH6A1	VSX2
	10	11(1)	58,260,496–58,260,907	Forward	–		PTP	LRRTM4
	11	11(2)	107,297,269–107,297,597	Forward	–		PSMD13	–
	12	15	36,723,446–36,723,809	Forward	SOX6	E3-E4 intron	SMAP	INSC
	13	18	8,775,609–8,776,056	Forward	MPHOSPH6	E2-E3 intron	HSD17B2	CDH13
	14	X(1)	91,193,469–91,194,016	Forward	–		ARAF	SYN1
	15	X(2)	80,581,077–80,581,395	Forward	–		PBDC1	MAGEE2

**Table 4:** Statistics of SNP and INDEL.

I.D. (DNA)	The number of SNP	The number of INDEL
Wild Type (No transgene)	8,113,244	1,141,867
SNU-SB-1 (SB-pCAG-YFP)	8,194,444	1,156,313
SNU-PB-1 (PB-pCAG-rox-GFP-rox-RFP)	8,146,673	1,166,927
SNU-PB-2 (PB-pβ-casein-hIL2-pCAG-GFP)	8,127,879	1,142,798

**Table 5:** Statistics of SNP and INDEL in comparison of transgenic cattle to wild type.

Type	SNP	INS	DEL	Total
All variants	2,016,456	90,135	108,401	2,214,992
High impact only#1	177	65	73	315

^#1^Using cattle genomes (UMD3.1.78), SnpEff was applied to predict high-impact single nucleotide variant (SNV) resulting in gain/loss stop codon, frame-shift, splice site changes (donor or acceptor) or loss of start codon in these elite natural variants.

**Table 6:** Relative telomere lengths in cattle.

I.D. (Age; DNA; Resource)	Estimated telomere length
Wild Type (24 months old; No transgene; Blood)	5.68661
SNU-SB-1 (10 months old; SB-pCAG-YFP; Blood)	6.59096
SNU-PB-1 (4 months old; PB-pCAG-rox-GFP-rox-RFP; Blood)	7.26370
SNU-PB-1 (4 months old; PB-pCAG-rox-GFP-rox-RFP; Primary cells)	7.61535
SNU-PB-2 (2 months old; PB-pβ -casein-hIL2-pCAG-GFP; Blood)	6.98291
