# Supplementary material for: Efficient generation of transgenic cattle using the DNA transposon and their analysis by next-generation sequencing
Source: Sci Rep. 2016 Jun 21;6:27185. doi: 10.1038/srep27185 (PMC4914850; doi:10.1038/srep27185)
Supplement: Supplementary Information [file srep27185-s1.pdf]

Supplementary Figures

Efficient generation of transgenic cattle using the DNA transposon and their analysis by next-generation sequencing

Soo-Young Yum[1,†], Song-Jeon Lee[2,†], Hyun-Min Kim[4,†], Woo-Jae Choi[1], Ji-Hyun Park[1], Won-Wu Lee[2], Hee-Soo Kim[2], Hyeong-Jong Kim[2], Seong-Hun Bae[2], Je-Hyeong Lee[2], Joo-Yeong Moon[2], Ji-Hyun Lee[1], Choong-Il Lee[1,3], Bong-Jun Son[4,] Sang-Hoon Song[4], Su-Min Ji[4], Seong-Jin Kim[4], Goo Jang[1,5,*]

[1]Department of Theriogenology, College of Veterinary Medicine and the Research Institute of Veterinary Science, Seoul National University, Republic of Korea, 08826

[2]Embryo Research Center, Seoul Milk Coop, Gyeonggi-do, Republic of Korea, 12528

[3]Department of Chemistry, College of Natural Science, Seoul National University, Republic of Korea, 08826

[4]TheragenEtex BiO Institute, Advanced Institutes of Convergence Technology, Kwanggyo Technovalley, Suwon, Republic of Korea, 16229

[5]Emergence Center for Food-Medicine Personalized Therapy System, Advanced Institutes of Convergence Technology, Seoul National University, Gyeonggi-do, Republic of Korea, 16229

Supplementary Figure legends

Supplementary Figure 1. Illustration of DNAs used for this study. (A) YFP expressing DNAs using Sleeping Beauty, (B) DNAs for before (upper) and after (lower) Dre recombination, (C) hIl2 expressing DNAs under beta-casein promoter. In each figures, size for arrow indicates the region of genomic amplified DNAs.

Supplementary Figure 2. GFP expression ratio after transfection of normal plasmids (NP), sleeping beauty (SB) and piggybac (PB) into bovine fibroblasts. After transfection, we counted the GFP positive cells at every 24 h (from 24 h to 144 h) and recorded as percentage units.

Supplementary Figure 3. Autopsy of the dead transgenic cattle (SNU-PB-3). Pictures of the head with fluorescence (A) or brightness (B). The pictures of the intestine (C), heart (D), spleen (E) and liver (F) under the fluorescence.

Supplementary Figure 4. The pictures of four transgenic cattle. (A) Transgenic cattle (SNU-PB-4): Pictures of the head at neonate (a) and at one month later (b), Strong GFP expression in the nose (c-arrow), eyes (d-arrow) and tongue (e-arrow); (B) Picture of transgenic cattle (SNU-PB-6): the picture of neonate calf (a) and strong expression of GFP in the eyes (b); (C) Picture of transgenic cattle (SNU-PB-7): the picture of neonate calf (a) and strong expression of GFP in the eyes (b); (D) Picture of transgenic cattle (SNU-PB-8): the picture of neonate calf (a) and strong expression of GFP in the eyes (b).

Supplementary Figure 5. Guanine Nucleotide Binding Protein (G Protein), Alpha Inhibiting Activity Polypeptide 1 (GNAI1) expression in various organ. (A) Illustration of transgene insertion in between exon 1 and 2; (B) GNAI1 expression in various cell or tissues.

Supplementary Figure 6. GFP disruption and knock-in of puromycin resistance gene using CRISPR/Cas9 in fibroblasts from a transgenic cattle (SNU-PB-2). (A) Number of colonies after transfection with sgRNA for GFP, Cas9 and donor DNA, (B) Illustration of GFP disruption and knock-in into the fibroblasts from a transgenic cattle.

Supplementary Figure 7. YFP expression in the oocytes and endometrial cells. (A) Picture of nine oocytes after superovulating the transgenic cattle (SNU-SB-1), (B) Transgene expression in genomic DNA from the oocytes, (C) Sequencing validation of transgene PCR amplicons, (D) Confocal images of YFP expression in control fibroblasts (upper panel) and endometrial cells (lower panel).

Supplementary Figure 8. Lists of original gel images used for main figures.

Supplementary Figure 1

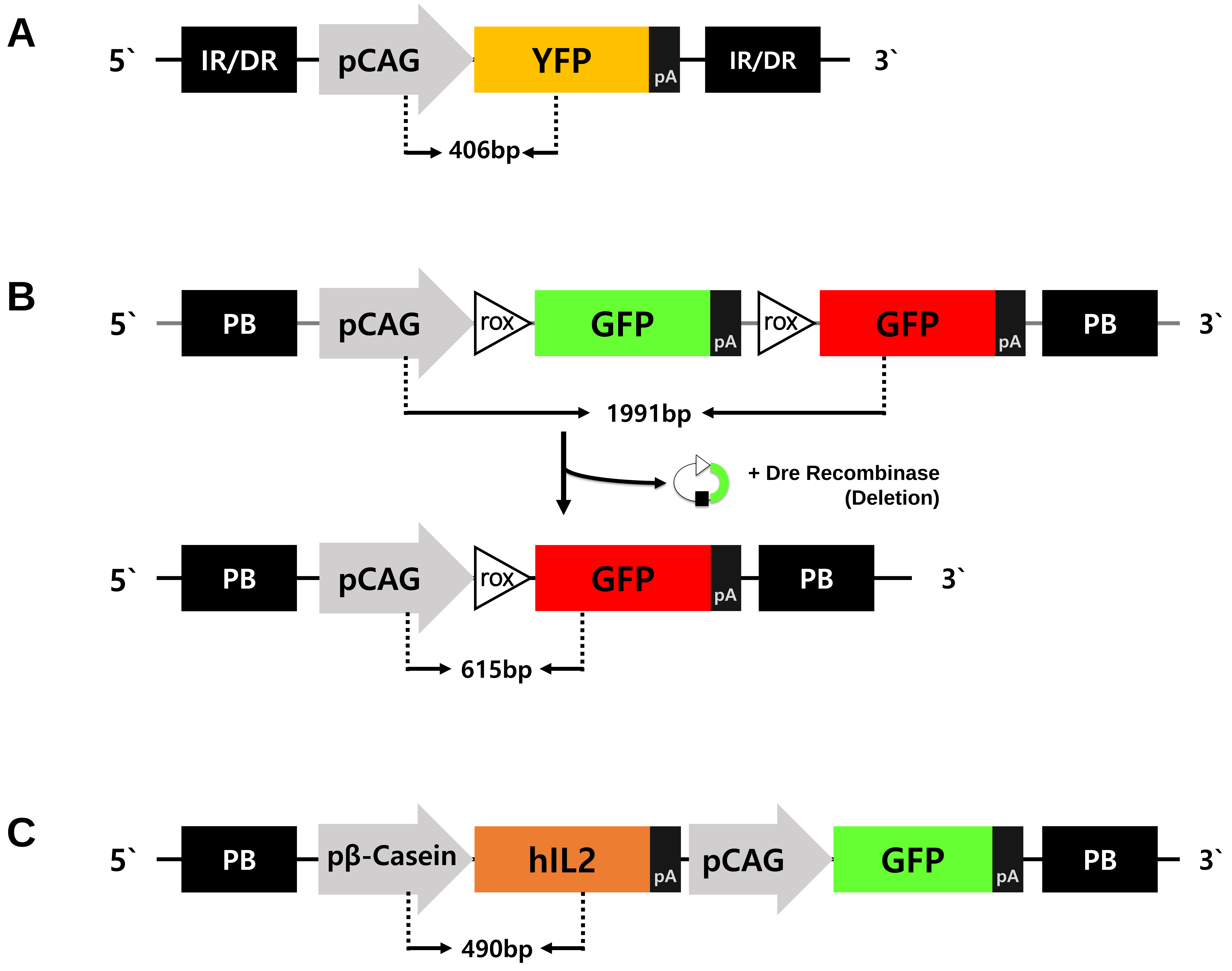

Supplementary Figure 2

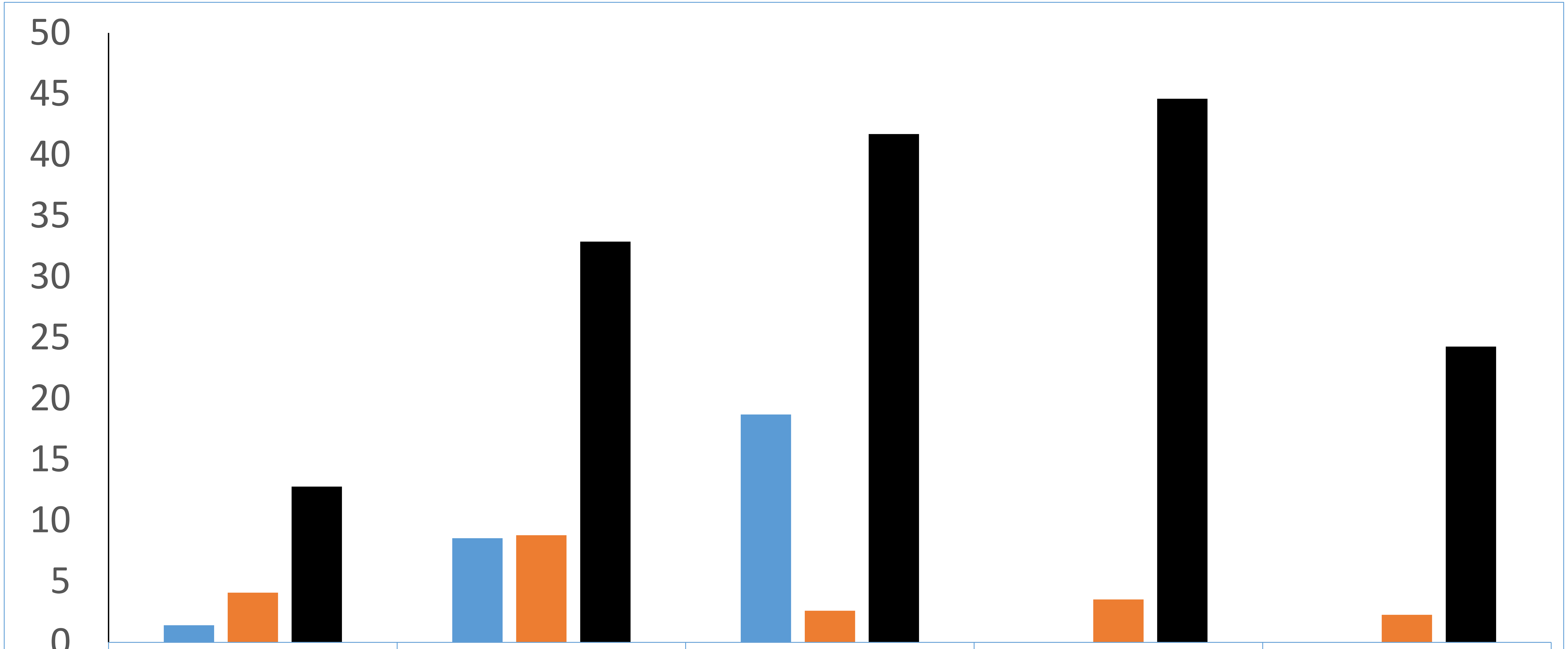

	24h	48h	72h	96h	144h
NP	1.41	8.55	18.7	0	0
SB	4.075	8.795	2.6	3.52	2.26
PB	12.785	32.875	41.7	44.6	24.26

Supplementary Figure 3

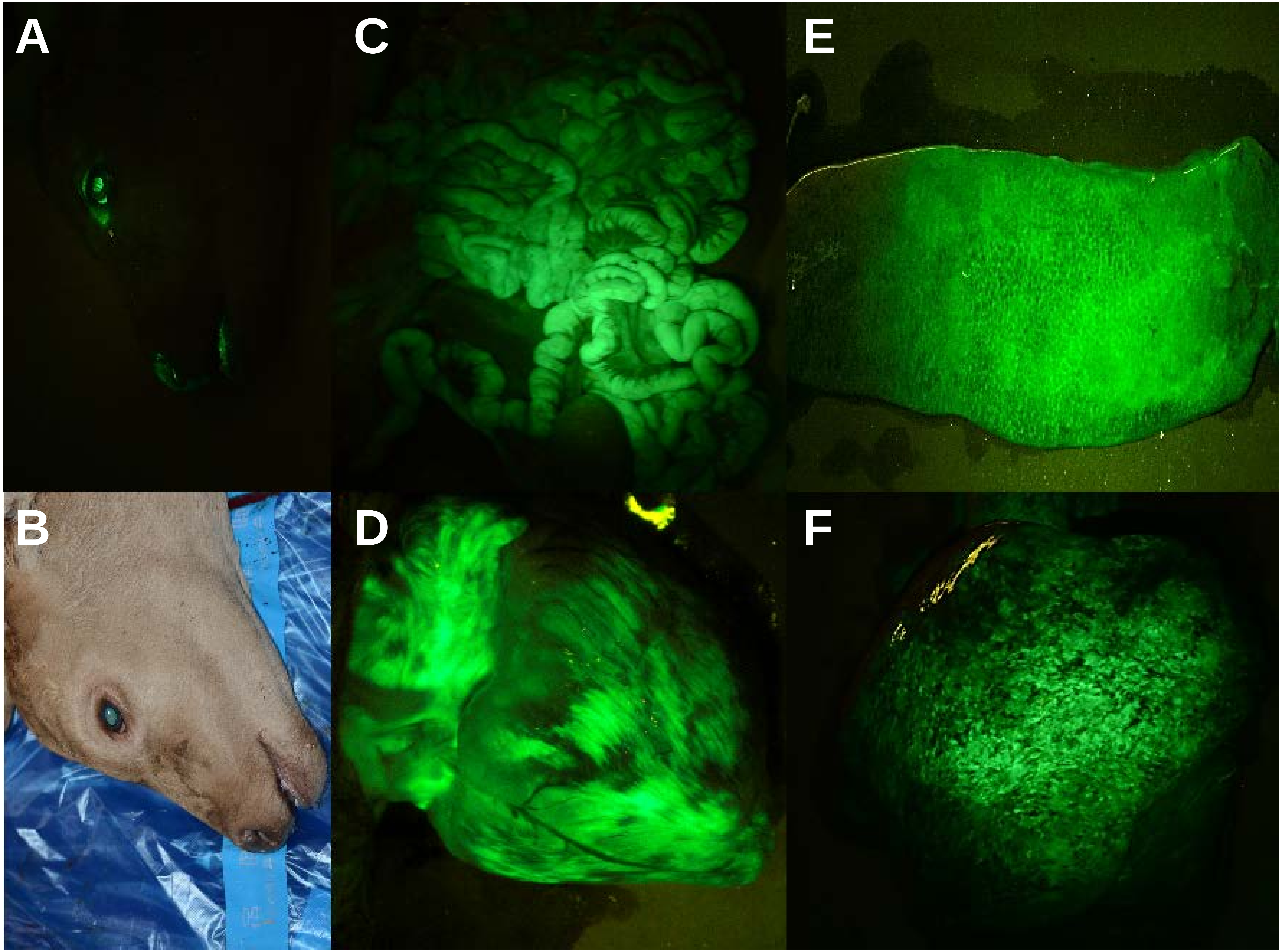

Supplementary Figure 4

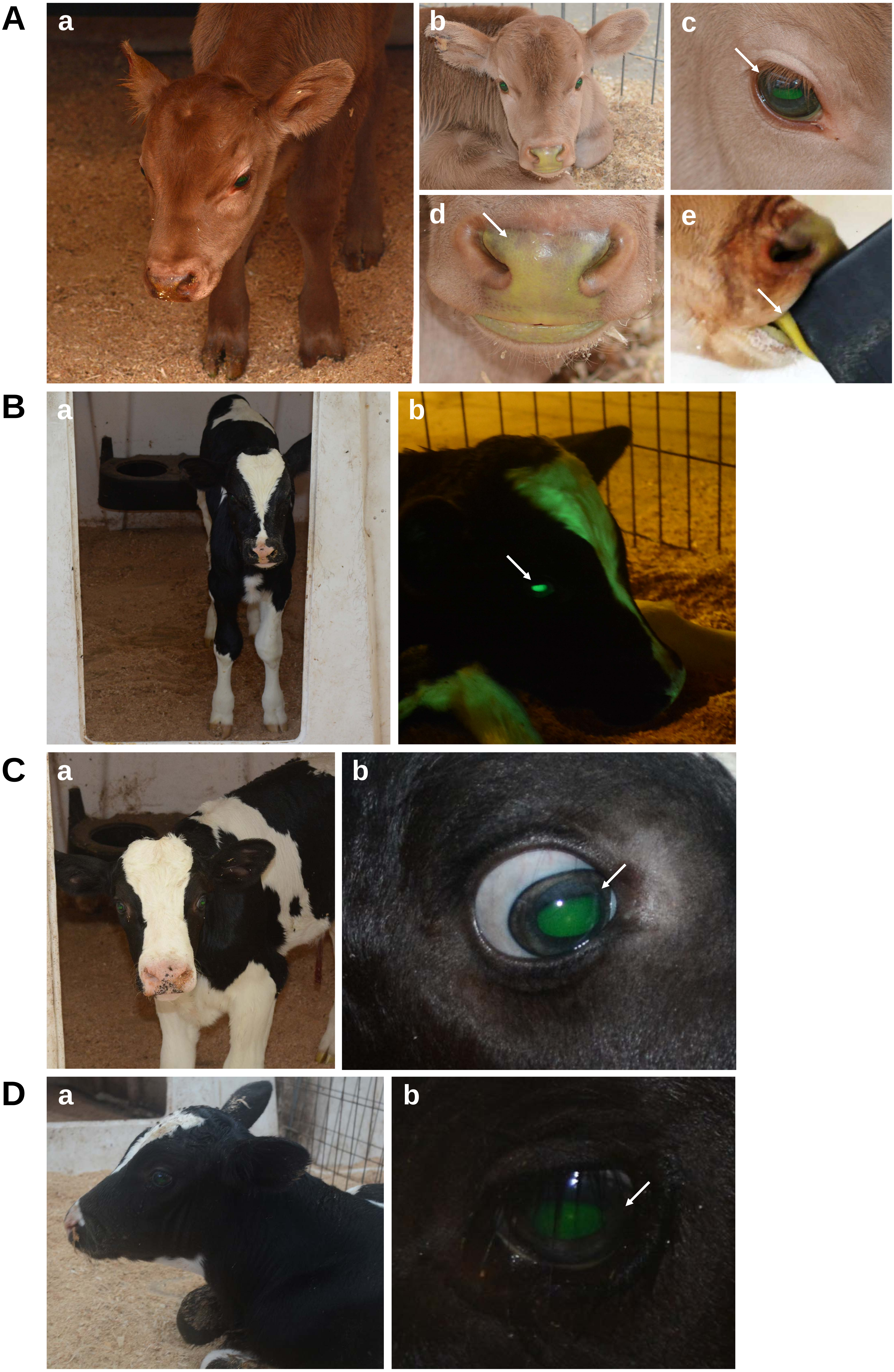

Supplementary Figure 5

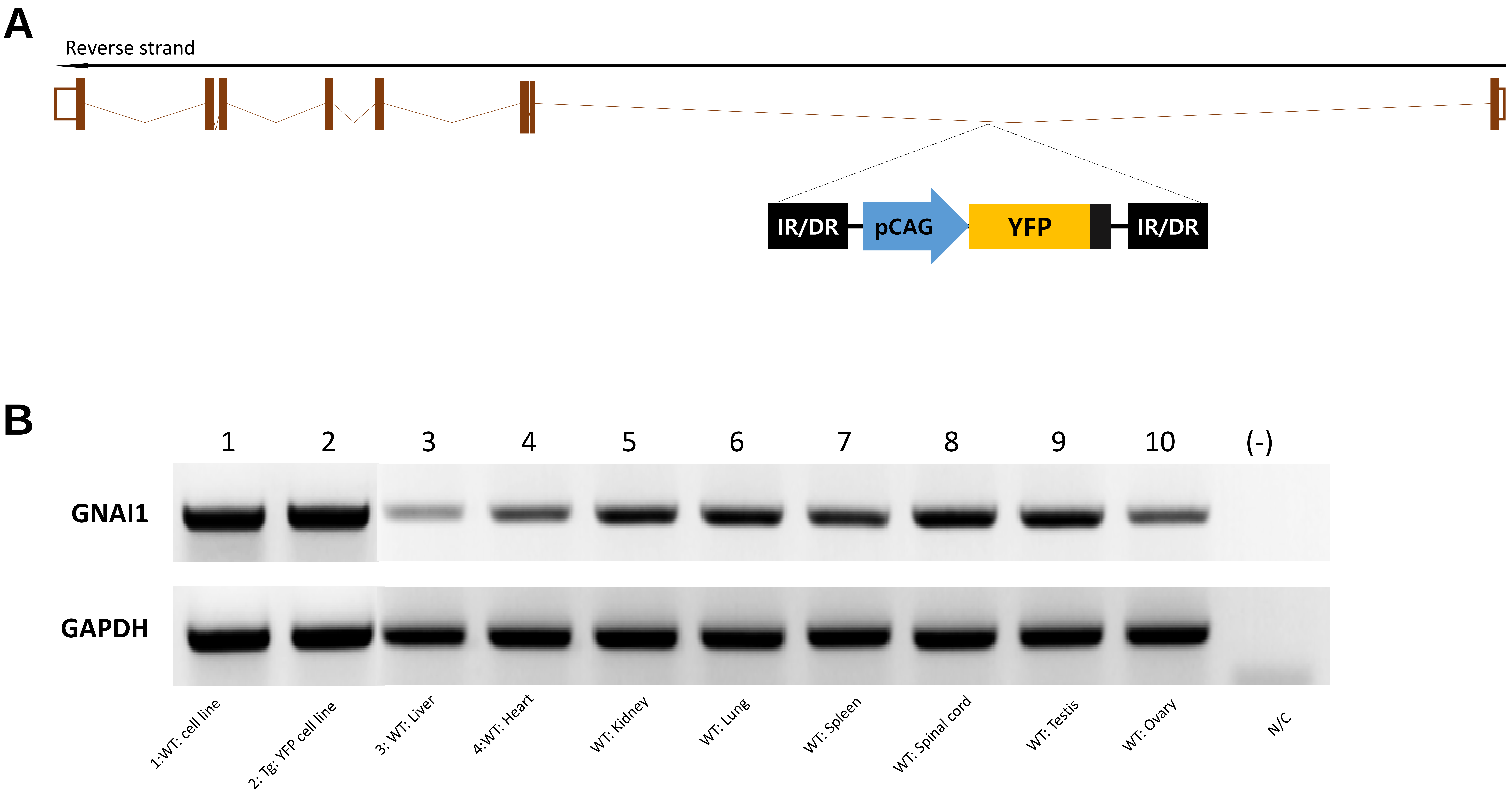

Supplementary Figure 6

A

	Wild Type	GFP guide RNA/Cas9	Donor	GFP guide RNA/Cas9/Donor
**Colony Numbers**	**0**	**0**	**0**	**>20**

Puromycin selection 6 day

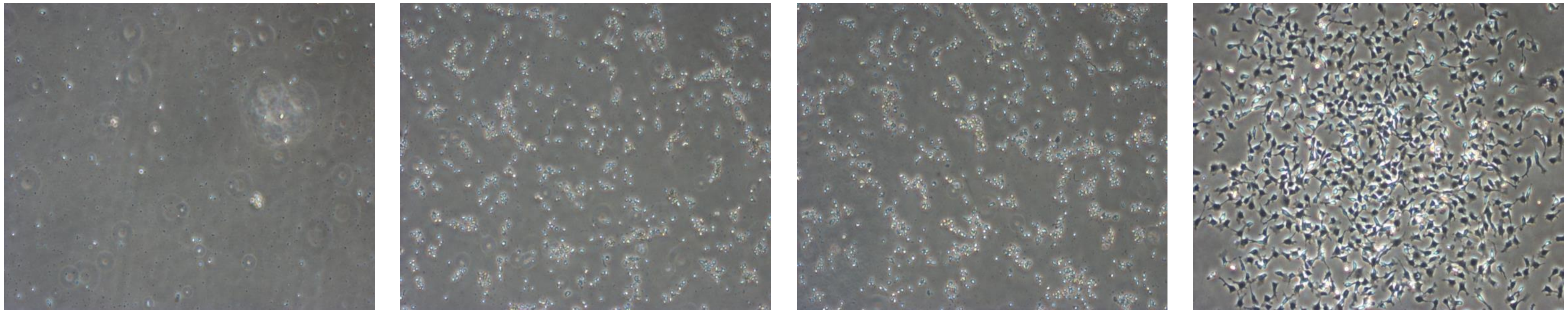

B

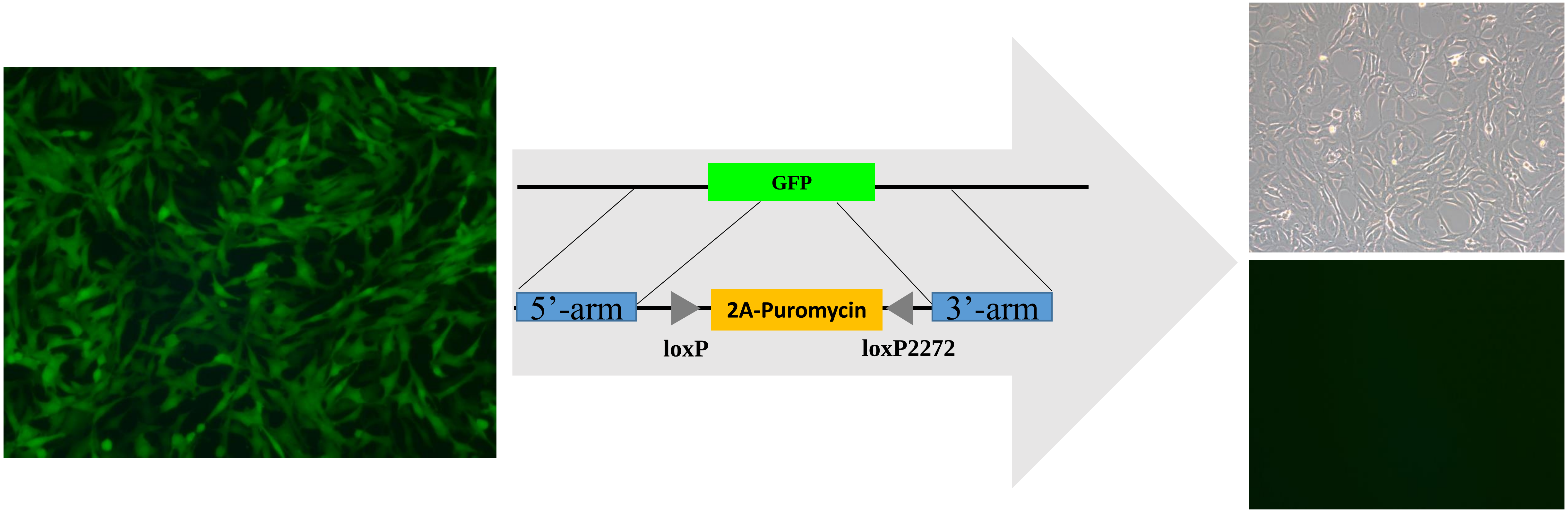

Supplementary Figure 7

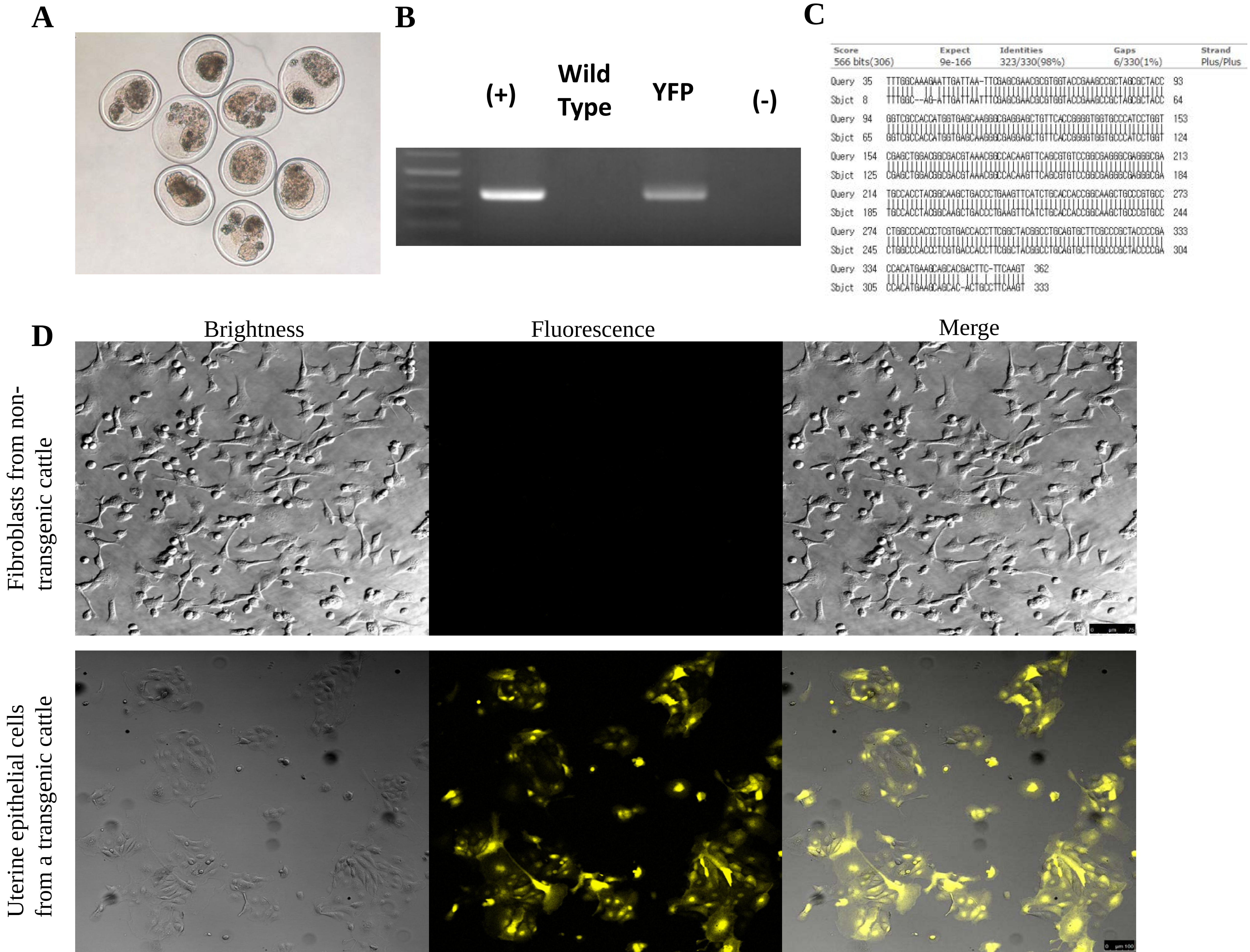

Supplementary Figure 8

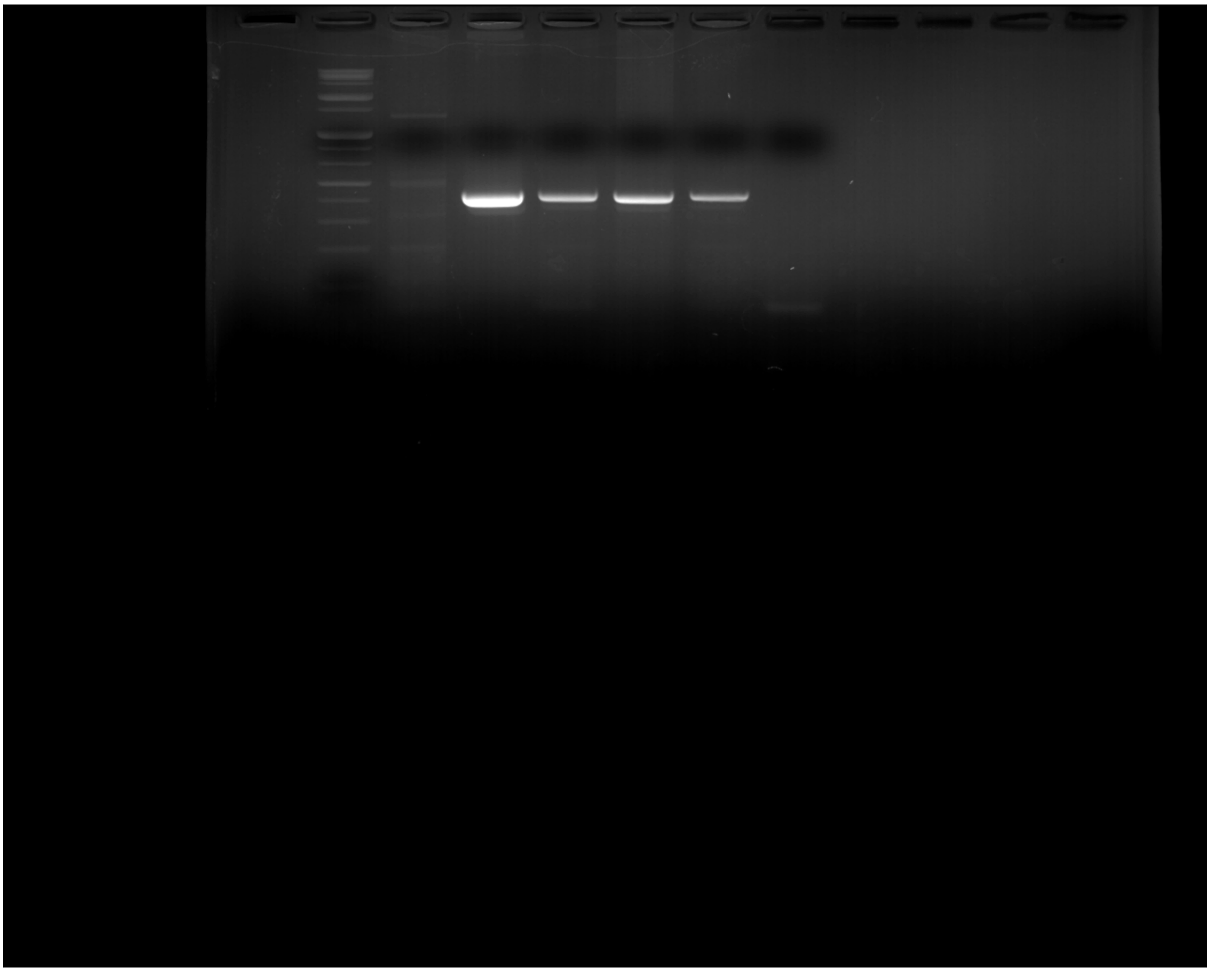

S-Figure 8-1. Original image of Figure 2g.

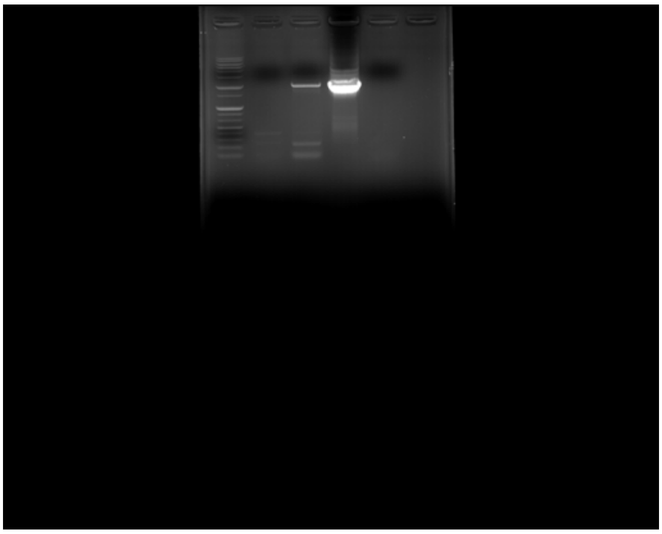

S-Figure 8-2. Original image of Figure 3h.

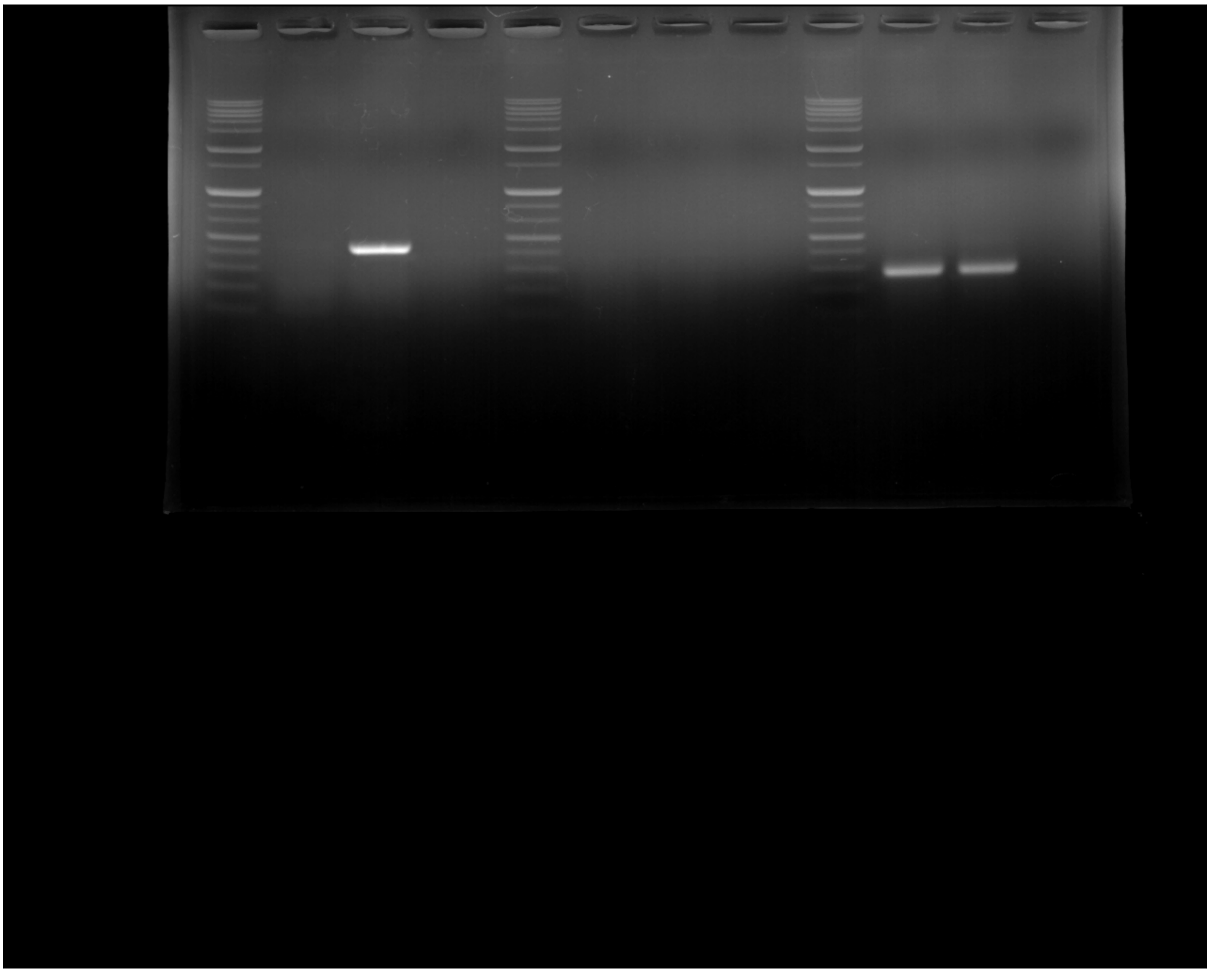

S-Figure 8-1. Original image of Figure 3i.

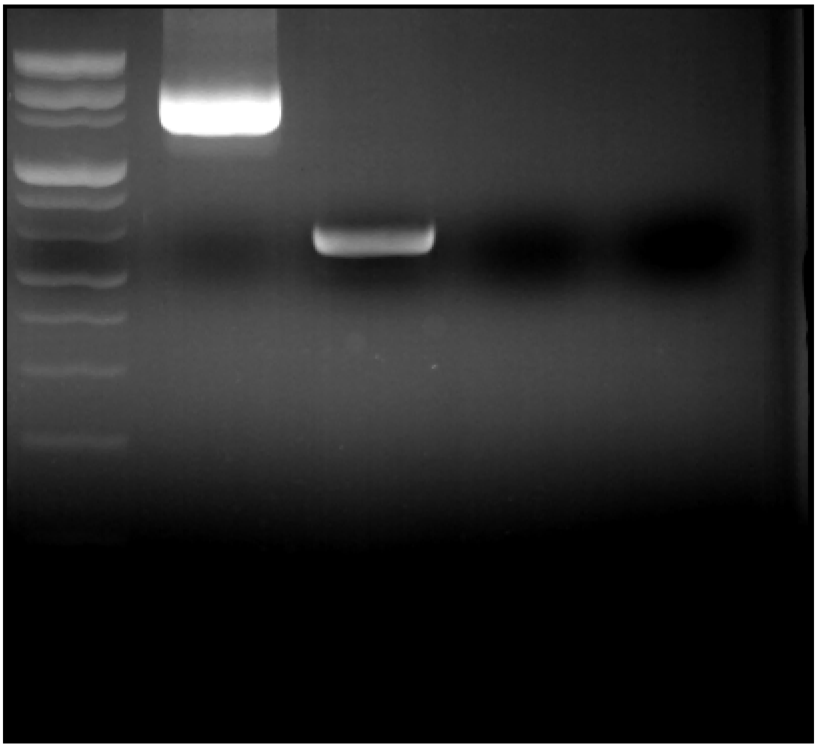

S-Figure 8-4. Original image of Figure 3j.

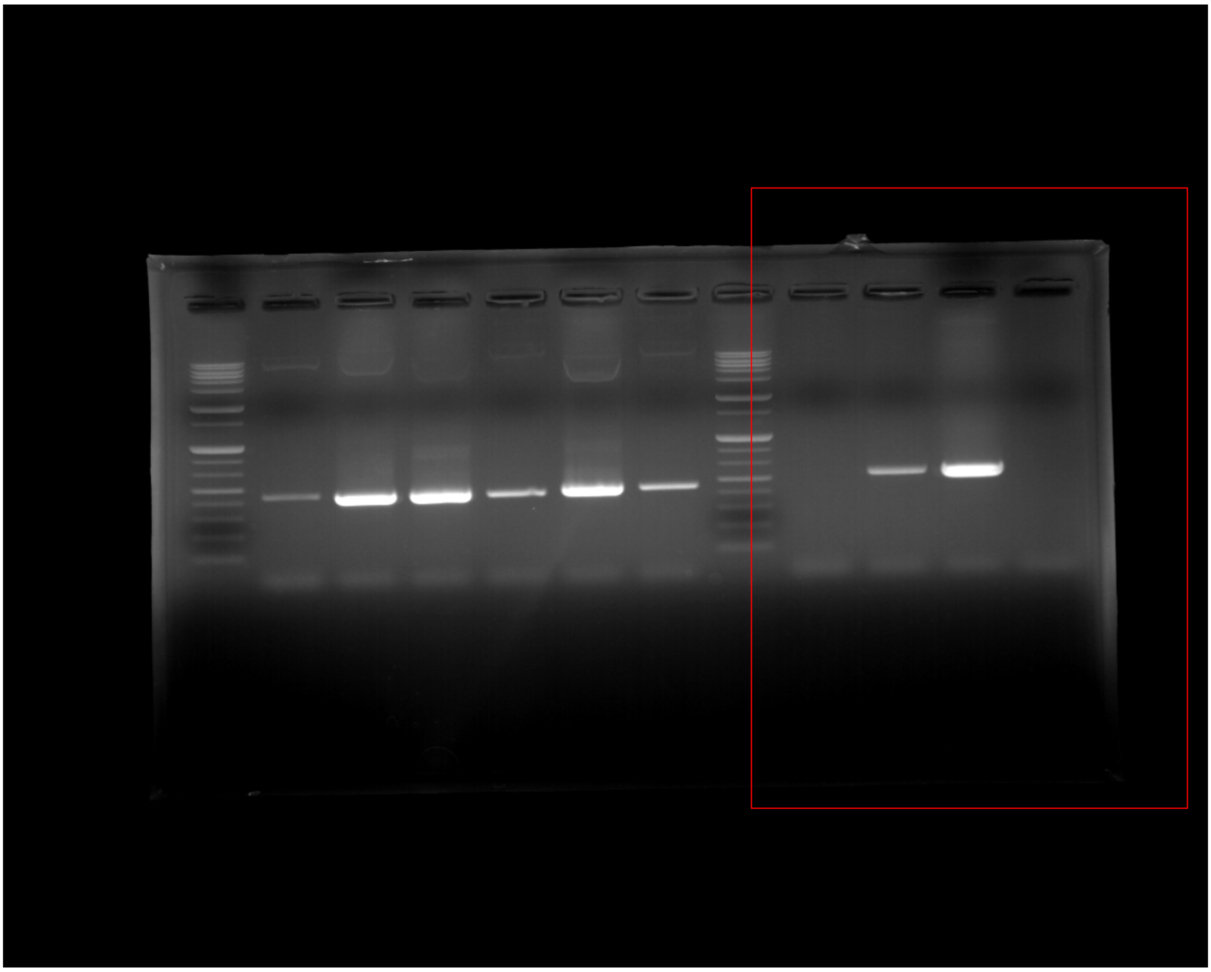

S-Figure 8-5. Original Image of Figure 4h.

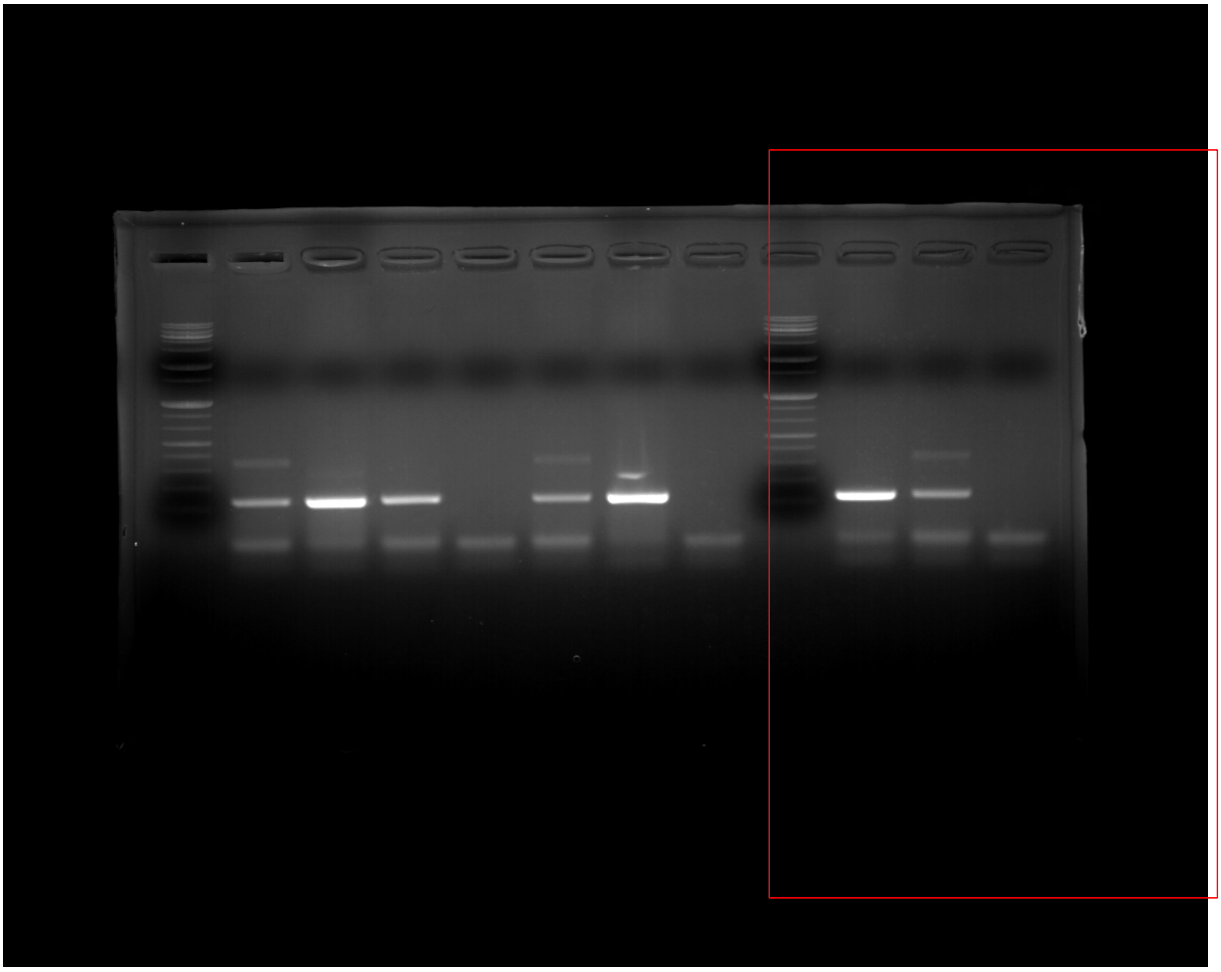

S-Figure 8-6. Original image of figure 4i (GAPDH).

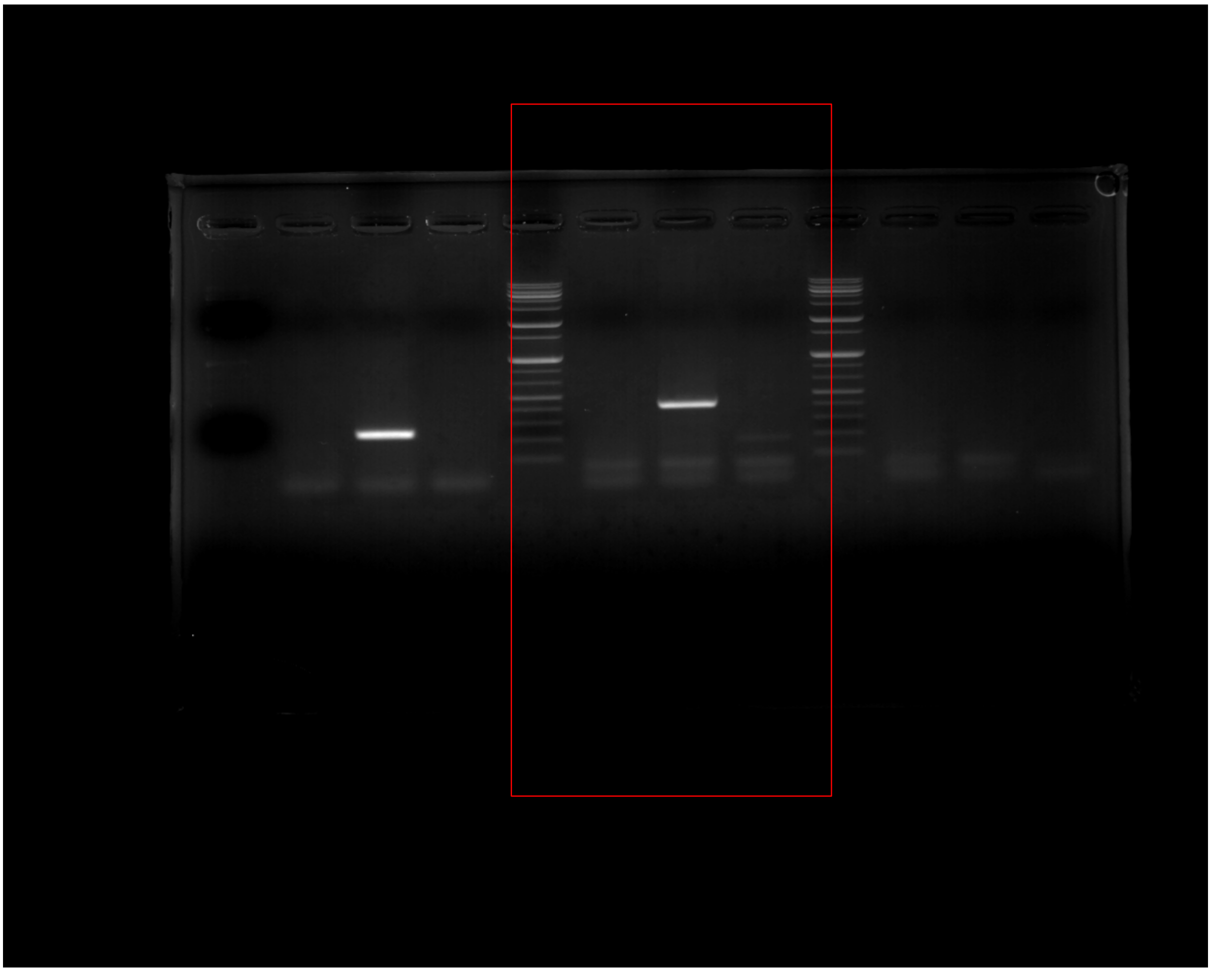

S-Figure 8-7. Original image of figure 4i (GFP).

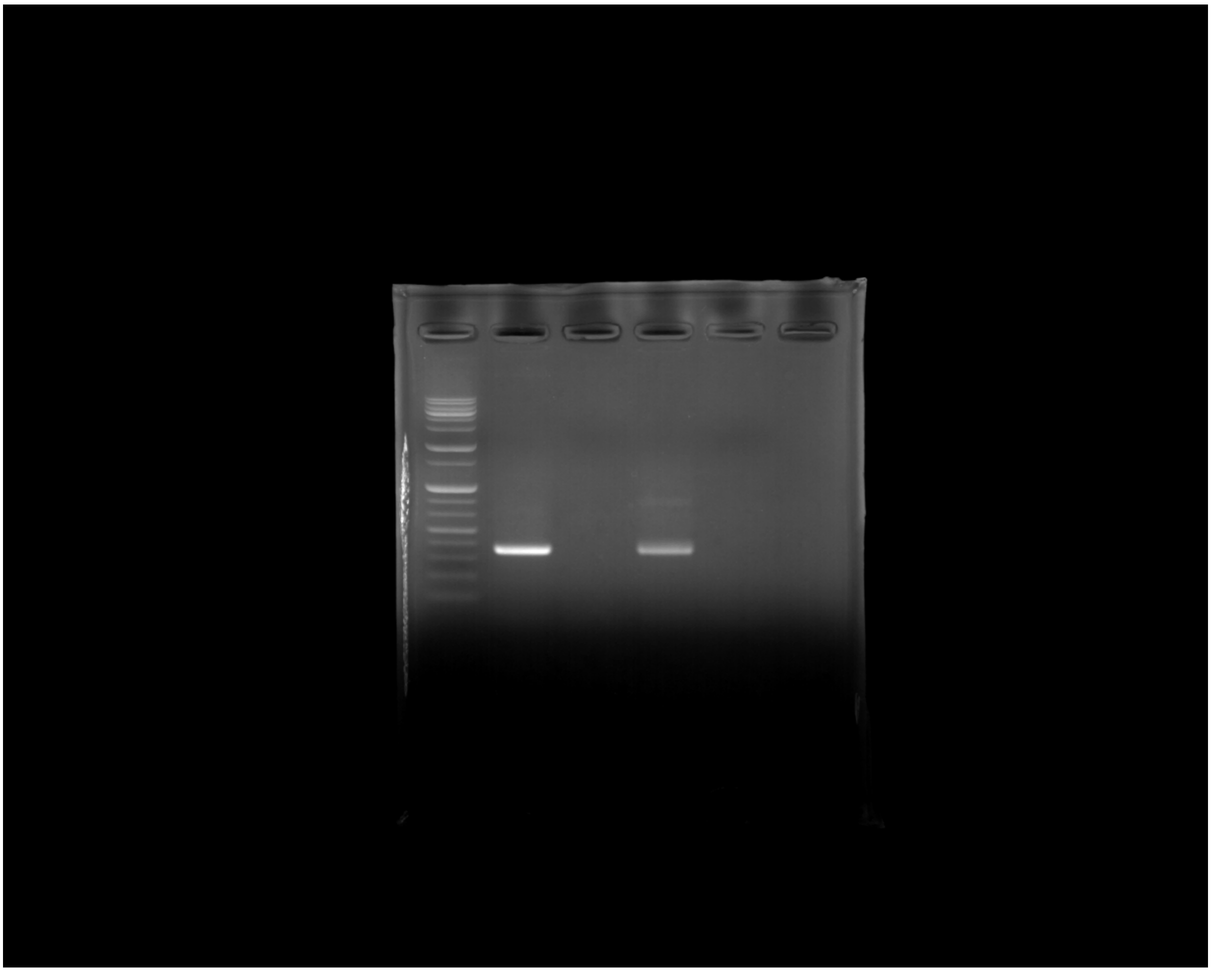

S-Figure 8-8. Original image of Supplementary-figure 7B.
